# Supplementary material for: Nanobodies for the Early Detection of Ovarian Cancer
Source: Int J Mol Sci. 2022 Nov 8;23(22):13687. doi: 10.3390/ijms232213687 (PMC9691119; doi:10.3390/ijms232213687)
Supplement: Supplementary file 1 [file ijms-23-13687-s001.zip › ijms-1981861-supplementary.pdf]

Nanobodies for the early detection of ovarian cancer

Electronic supplementary information

Lan-Huong Tran[1*], Geert-Jan Graulus[1*], Cécile Vincke[3], Natalia Smiejkowska[3], Anne Kindt[3], Nick Devoogdt[4], Serge Muyldermans[3], Peter Adriaensens[1,2], Wanda Guedens[1,†]

[1] Biomolecule Design Group, Institute for Materials Research (IMO), Hasselt University, Agoralaan - Building D, BE-3590 Diepenbeek, Belgium
[2] Analytical and Circular Chemistry, Institute for Materials Research (IMO), Hasselt University, Agoralaan - Building D, BE-3590 Diepenbeek, Belgium
[3] Laboratory of Cellular and Molecular Immunology, Vrije Universiteit Brussel, Pleinlaan 2, B-1050 Brussels, Belgium
[4] In Vivo Cellular and Molecular Imaging Laboratory (ICMI), Vrije Universiteit Brussel, Pleinlaan 2, B-1050 Brussels, Belgium

* Both authors contributed equally to this paper
† Corresponding author: W. Guedens: wanda.guedens@uhasselt.be

I. Kinetic results

Table S1. Antigen binding affinity of HE4 Nbs as determined by ELISA and SPR. * = could not be determined from the experimental data because Nbs have a k_{off} which is too high for a proper curve fit.

Sequence Families	Nbs	Expression level (mg/L)	ELISA	SPR			
			K_D (nM)	k_{on} ($M^{-1}.s^{-1}$)	k_{off} (s^{-1})	K_D (nM)	Chi^2 (RU^2)
F1	HE4-8	4.8	28.7	1.8E6	28.9E-3	16.0	0.02
	HE4-10	2.1	23.3	2.0E6	34.2E-3	18.0	0.02
F2	HE4-3	1.7	353.9	*	*	*	*
	HE4-5	2.2	198.1	*	*	*	*
	HE4-9	1.9	*	*	*	*	*
	HE4-13	9.1	746.8	*	*	*	*
F3	HE4-1	4.4	4.6	5.3E6	1.9E-3	0.4	0.02
	HE4-6	7.7	4.6	3.2 E6	9.0E-3	2.8	0.01
F4	HE4-2	0.6	509.7	*	*	*	*
F5	HE4-4	0.2	6.8	5.2E6	3.1E-3	0.6	0.02
F6	HE4-7	5.9	48.2	1.1E6	36.3E-3	32.0	0.03
F7	HE4-11	7.4	16.0	0.4E6	2.6E-3	6.1	0.02
F8	HE4-12	3.3	*	*	*	*	*

Table S2. Antigen binding affinity of SLPI Nbs as determined by ELISA and SPR. * = could not be determined from the experimental data because Nbs have a k_{off} which is too high for a proper curve fit.

Sequence Families	Nbs	Expression level (mg/L)	ELISA	SPR			
			K_D (nM)	k_{on} ($M^{-1}.s^{-1}$)	k_{off} (s^{-1})	K_D (nM)	Chi^2 (RU^2)
F1	SLPI-6	25.2	3.4	1.6E6	101.0E-3	62.0	0.06
F2	SLPI-2	0.2	4.3	3.3E6	54.5E-3	16.0	0.14
F3	SLPI-1	20.9	4.1	1.3E6	56.4E-3	43.0	0.20
F4	SLPI-3	2.5	32.4	5.8E6	12.9E-3	220.0	0.03
F5	SLPI-7	0.3	2.0	1.9E6	3.6E-3	1.9	1.00
F8	SLPI-4	0.8	78.9	1.6E6	208.6E-3	130.0	0.07
F9	SLPI-9	0.1	4.0	2.5E6	56.3E-3	22.0	0.14
F10	SLPI-8	1.3	91.5	1.2E6	297.0E-3	240.0	0.07

Table S3. Antigen binding affinity of PGRN Nbs as determined by ELISA and SPR. * = could not be determined from the experimental data because Nbs have a k_{off} which is too high for a proper curve fit.

**Sequence Families**	**Nbs**	**Expression level (mg/L)**	**ELISA**	**SPR**			
			**K_D (nM)**	**k_{on} ($M^{-1}.s^{-1}$)**	**k_{off} (s^{-1})**	**K_D (nM)**	**Chi^2 (RU^2)**
F1	PGRN-1	10.6	768.0	*	*	*	*
F2	PGRN-2	10.0	198.0	1.4E5	14.1E-3	100.0	*
F3	PGRN-3	1.3	175.0	*	*	*	*
F4	PGRN-4	15.0	15.0	1.7E5	26.5E-3	160.0	0.07
F5	PGRN-5	10.5	2.7	1.0E5	3.4E-3	33.0	0.01
F6	PGRN-6	9.6	*	*	*	*	*
F7	PGRN-7	0.5	1.1	8.8E5	87.9E-3	1.0	0.20
F8	PGRN-8	3.3	2.1	5.2E5	9.1E-3	17.0	0.22
F9	PGRN-9	1.5	3.5	19.0E5	57.2E-3	30.0	0.36
F10	PGRN-10	14.0	122.0	*	*	*	*
F11	PGRN-11	11.3	1.8	2.6E5	2.0E-3	7.5	0.10
F12	PGRN-12	0.9	*	*	*	*	*

II. SPR results

HE4

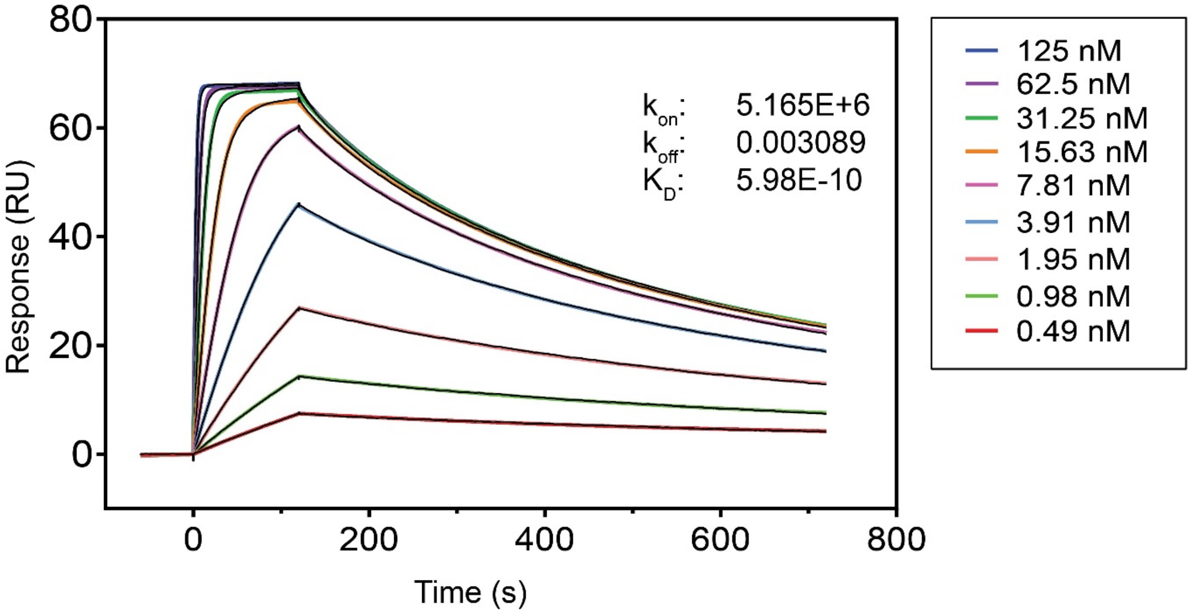

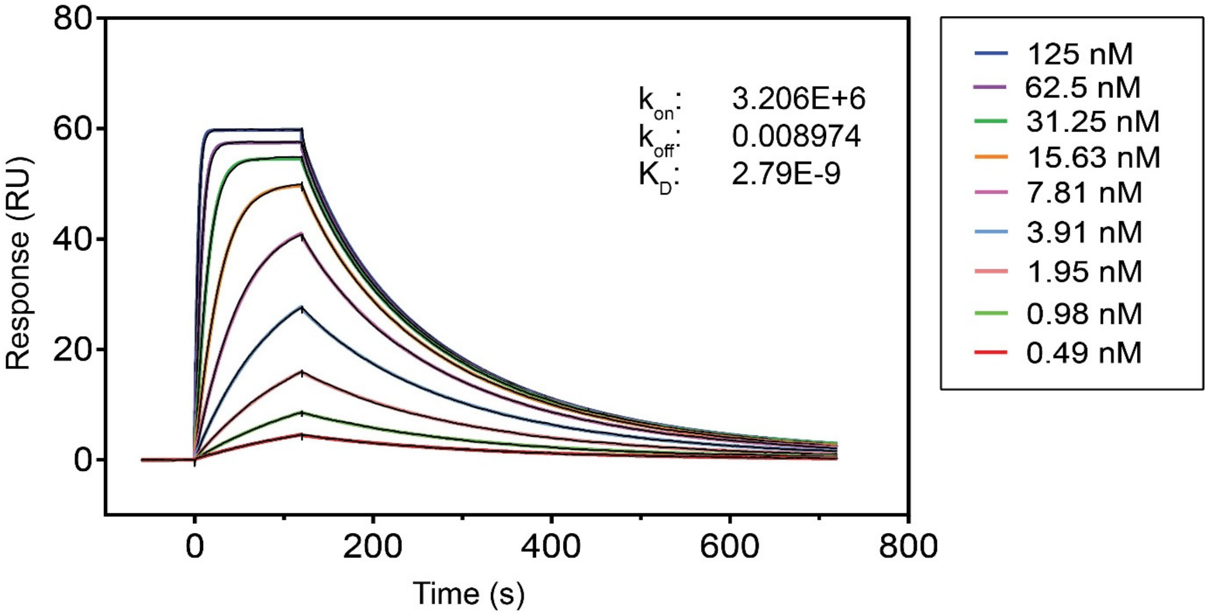

Figure S1. Antigen binding affinity of HE4 Nbs (k_{on} in M^{-1} s^{-1} and k_{off} in s^{-1}) as determined by SPR. HE4-4 (top) and HE4-6 (bottom).

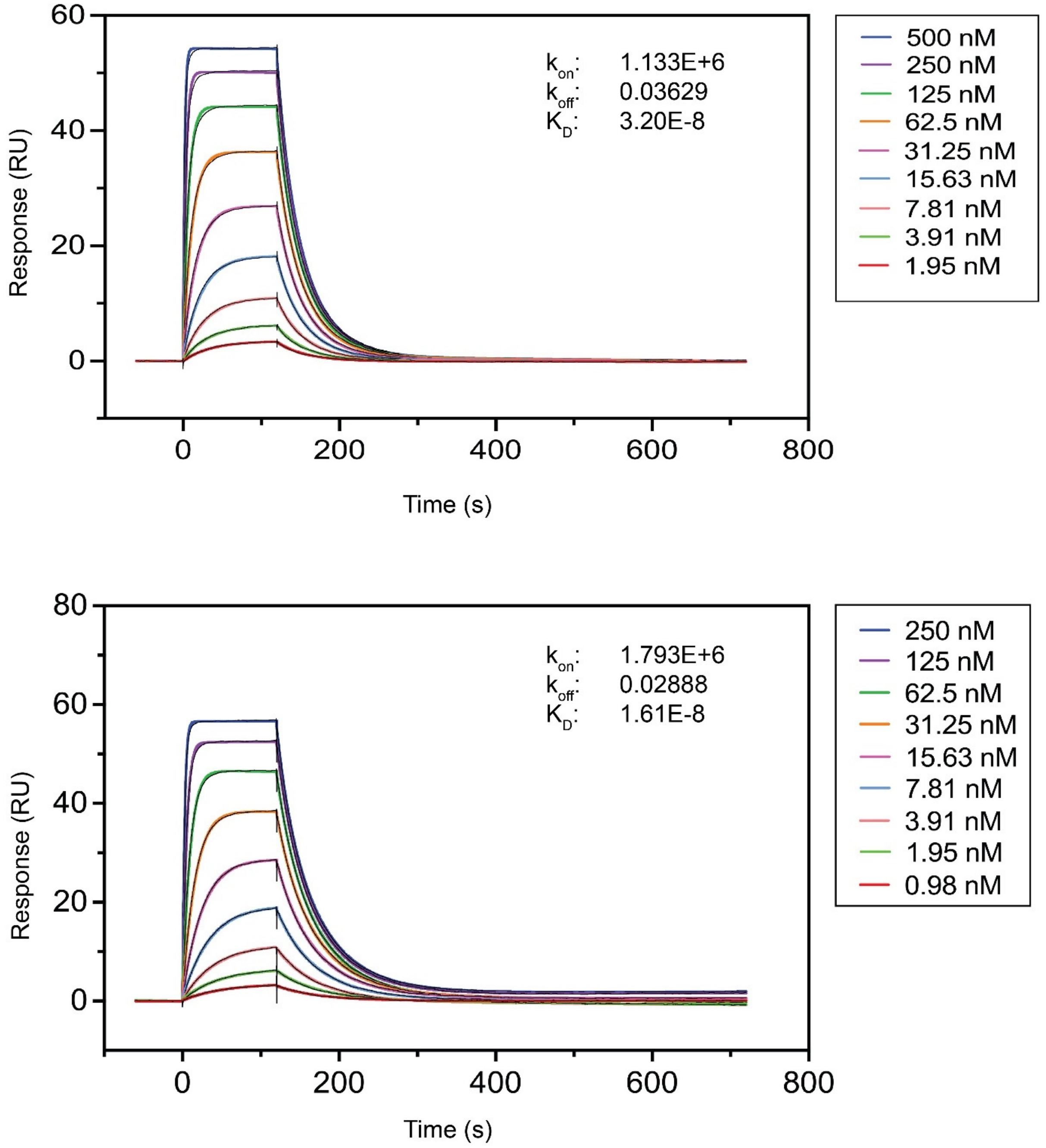

Figure S2. Antigen binding affinity of HE4 Nbs (k_{on} in M^{-1} s^{-1} and k_{off} in s^{-1}) as determined by SPR. HE4-7 (top) and HE4-8 (bottom).

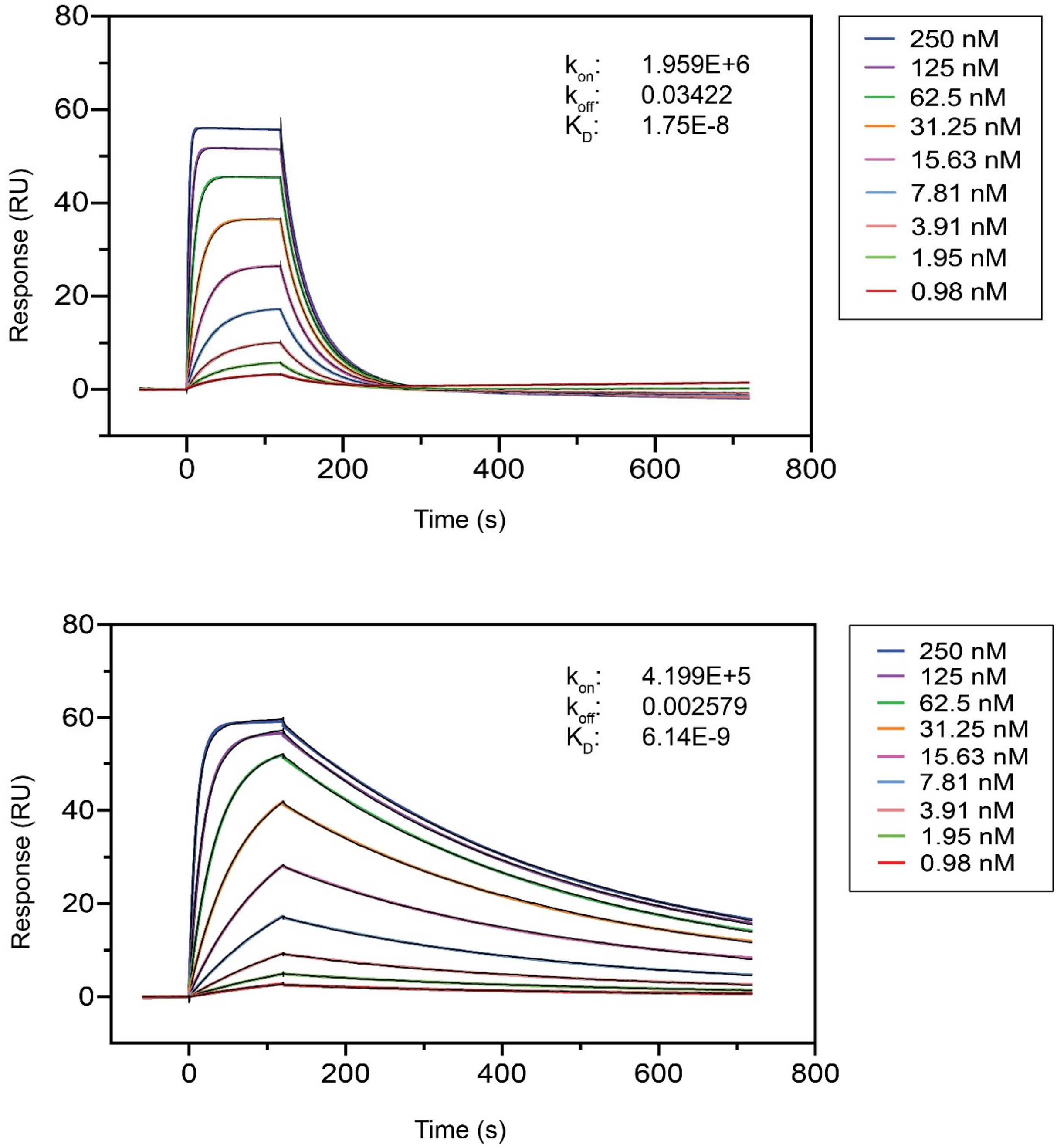

Figure S3. Antigen binding affinity of HE4 Nbs (k_{on} in M^{-1} s^{-1} and k_{off} in s^{-1}) as determined by SPR. HE4-10 (top) and HE4-11 (bottom).

SLPI

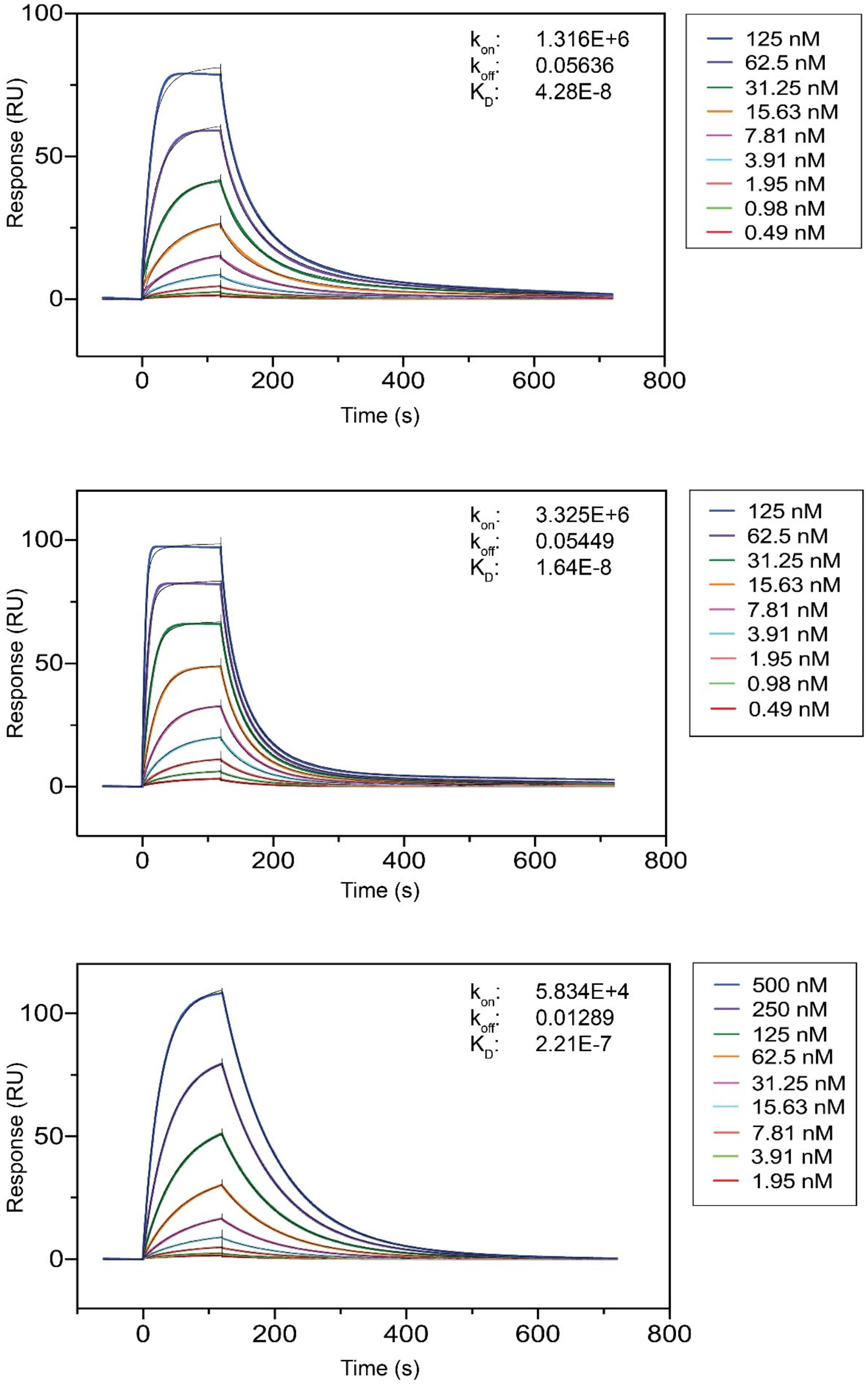

Figure S4. Antigen binding affinity of SLPI Nbs (k_{on} in M^{-1} s^{-1} and k_{off} in s^{-1}) as determined by SPR. SLPI-1 (top), SLPI-2 (middle) and SLPI-3 (bottom).

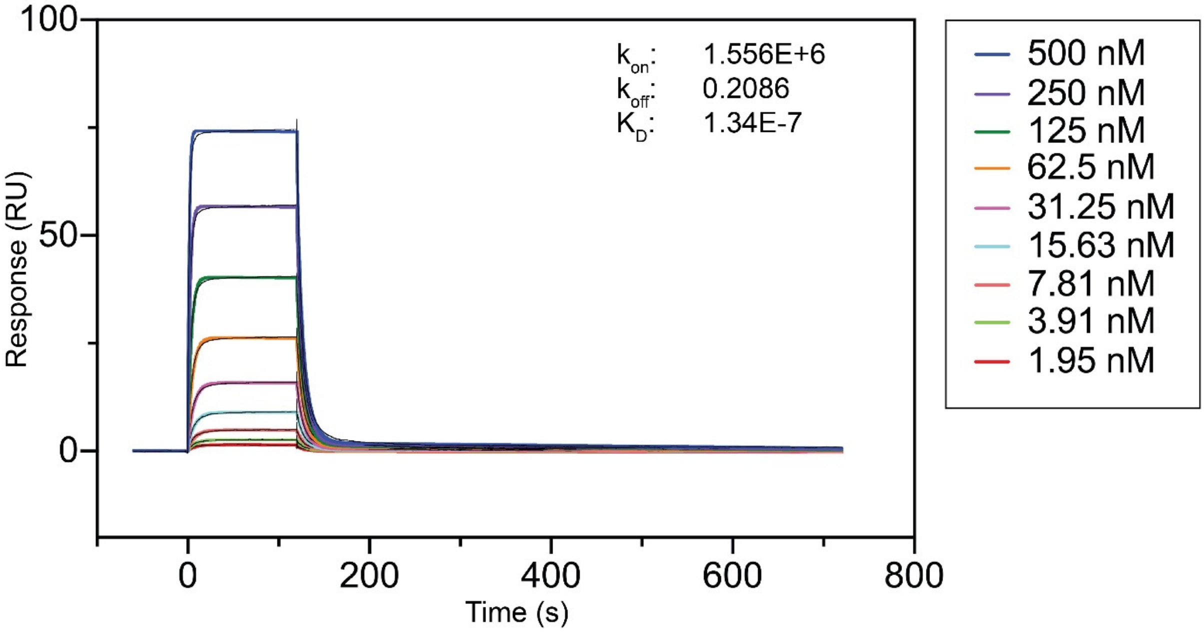

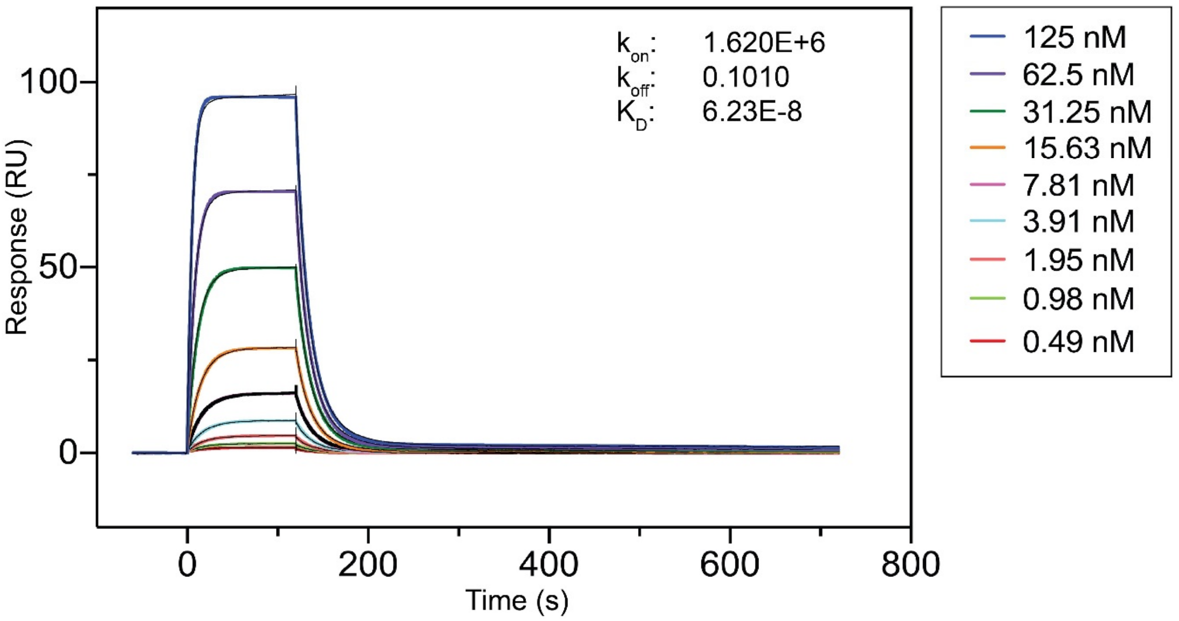

Figure S5. Antigen binding affinity of SLPI Nbs (k_{on} in M^{-1} s^{-1} and k_{off} in s^{-1}) as determined by SPR. SLPI-4 (top) and SLPI-6 (bottom).

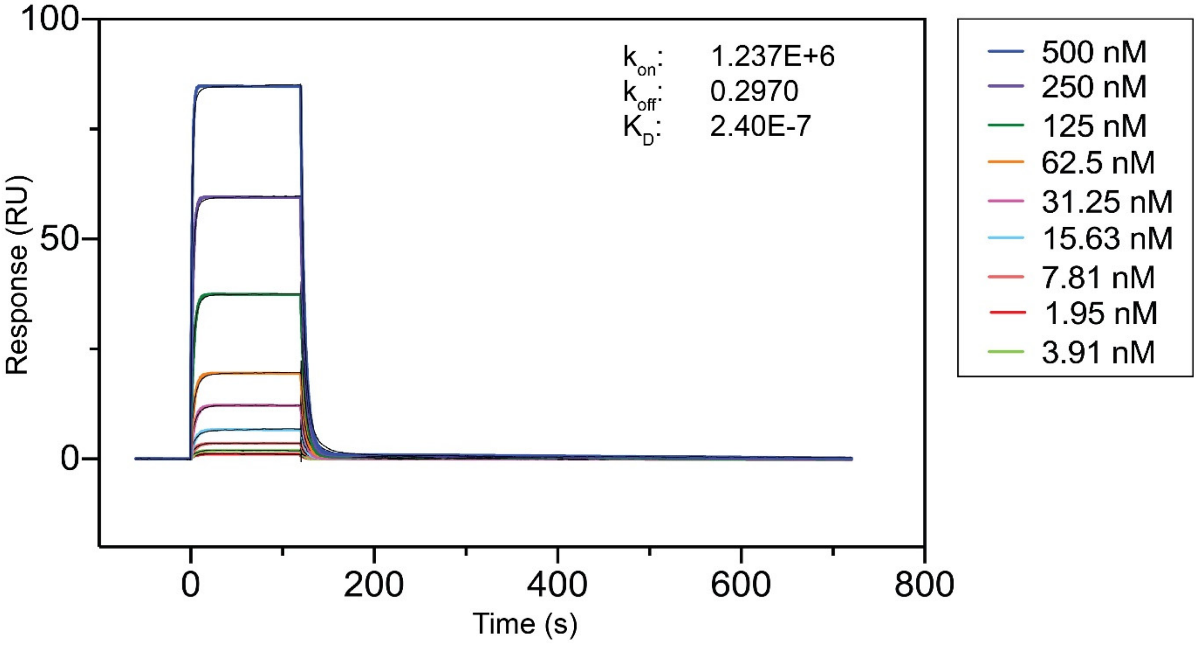

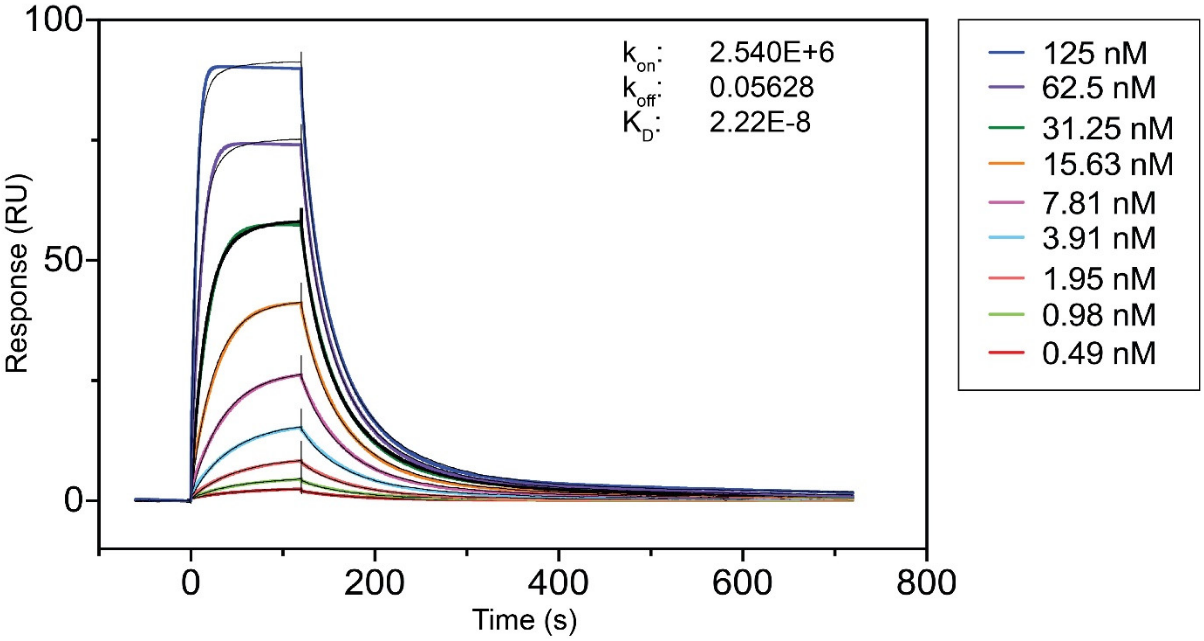

Figure S6. Antigen binding affinity of SLPI Nbs (k_{on} in M^{-1} s^{-1} and k_{off} in s^{-1}) as determined by SPR. SLPI-8 (top) and SLPI-9 (bottom).

PGRN

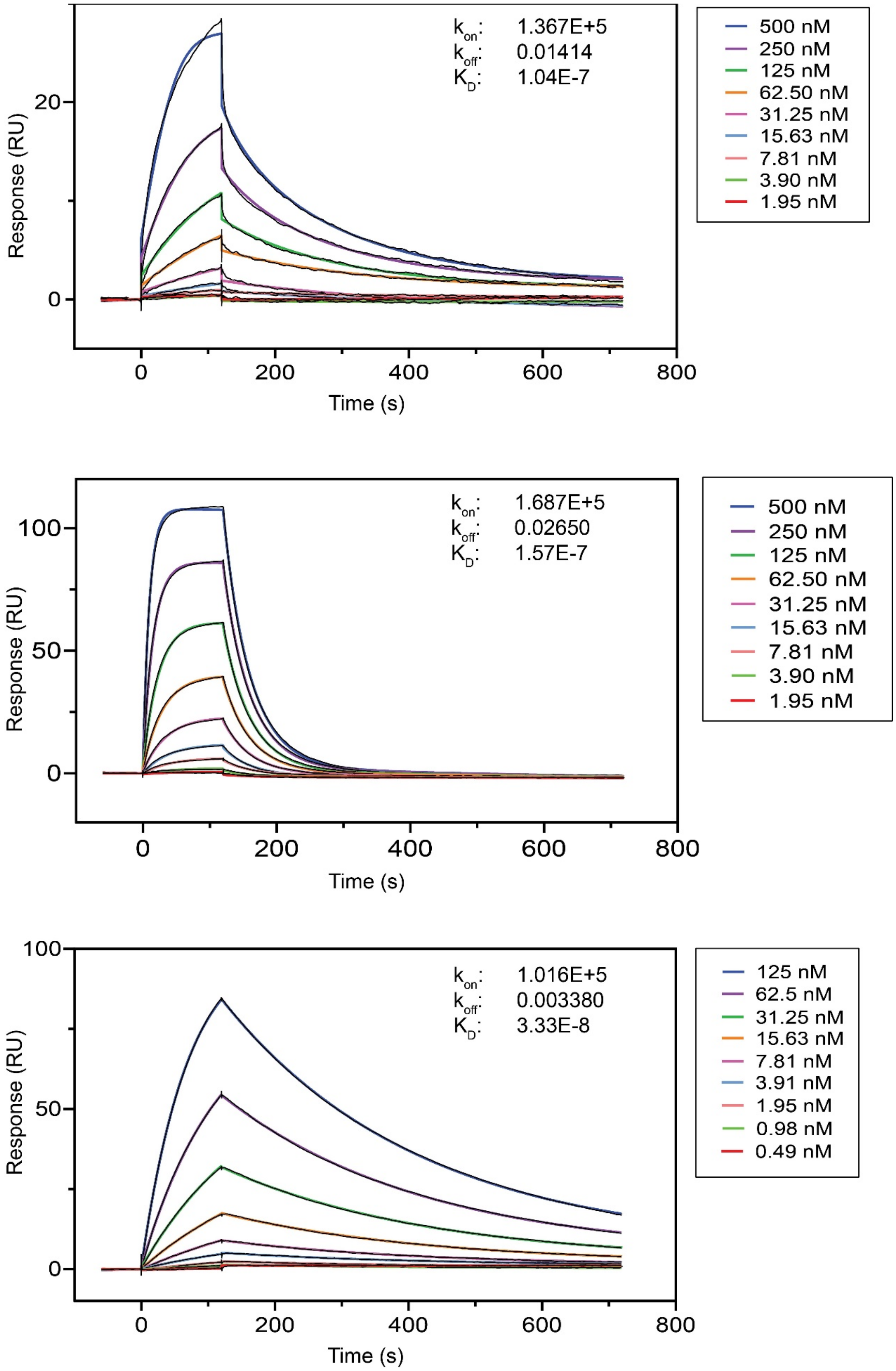

Figure S7. Antigen binding affinity of PGRN Nbs (k_{on} in M^{-1} s^{-1} and k_{off} in s^{-1}) as determined by SPR. PGRN-2 (top), PGRN-4 (middle) and PGRN-5 (bottom).

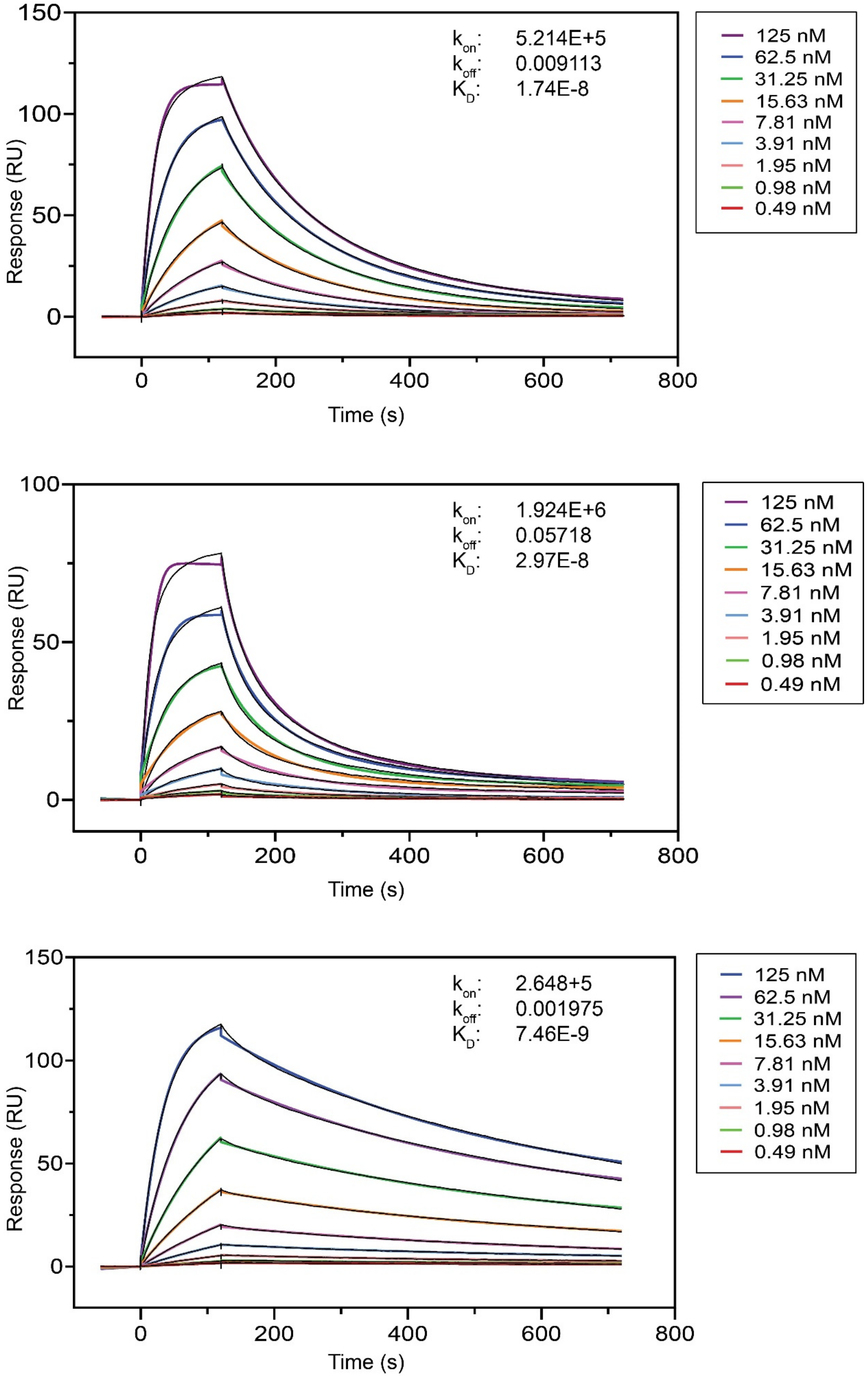

Figure S8. Antigen binding affinity of PGRN Nbs (k_{on} in M^{-1} s^{-1} and k_{off} in s^{-1}) as determined by SPR. PGRN-8 (top), PGRN-9 (middle) and PGRN-11 (bottom).

III. Epitope mapping

HE4

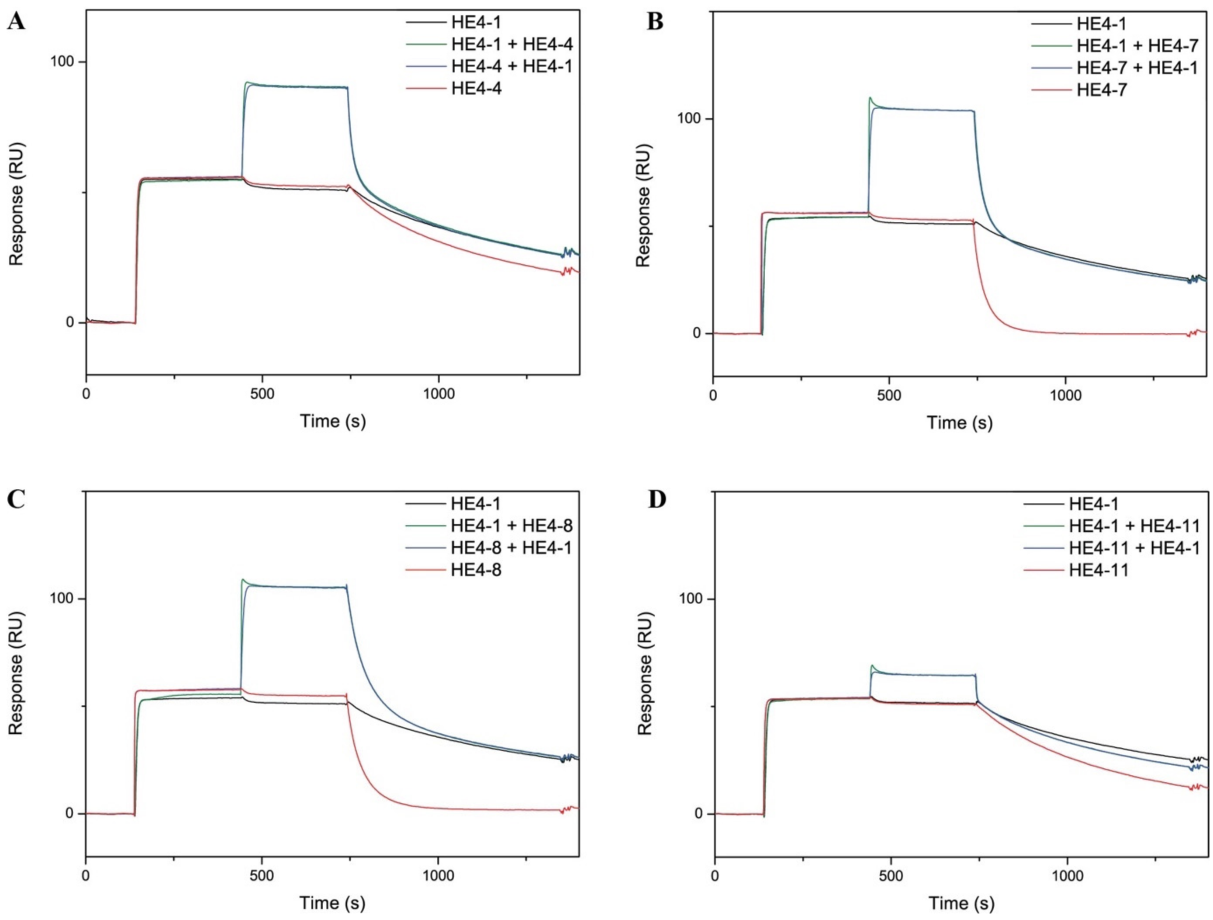

Figure S9. Competition in antigen binding affinity of HE4 Nbs as determined by epitope mapping. HE4-1 with HE4-4 (A), HE4-7 (B), HE4-8 (C) and HE4-11 (D).

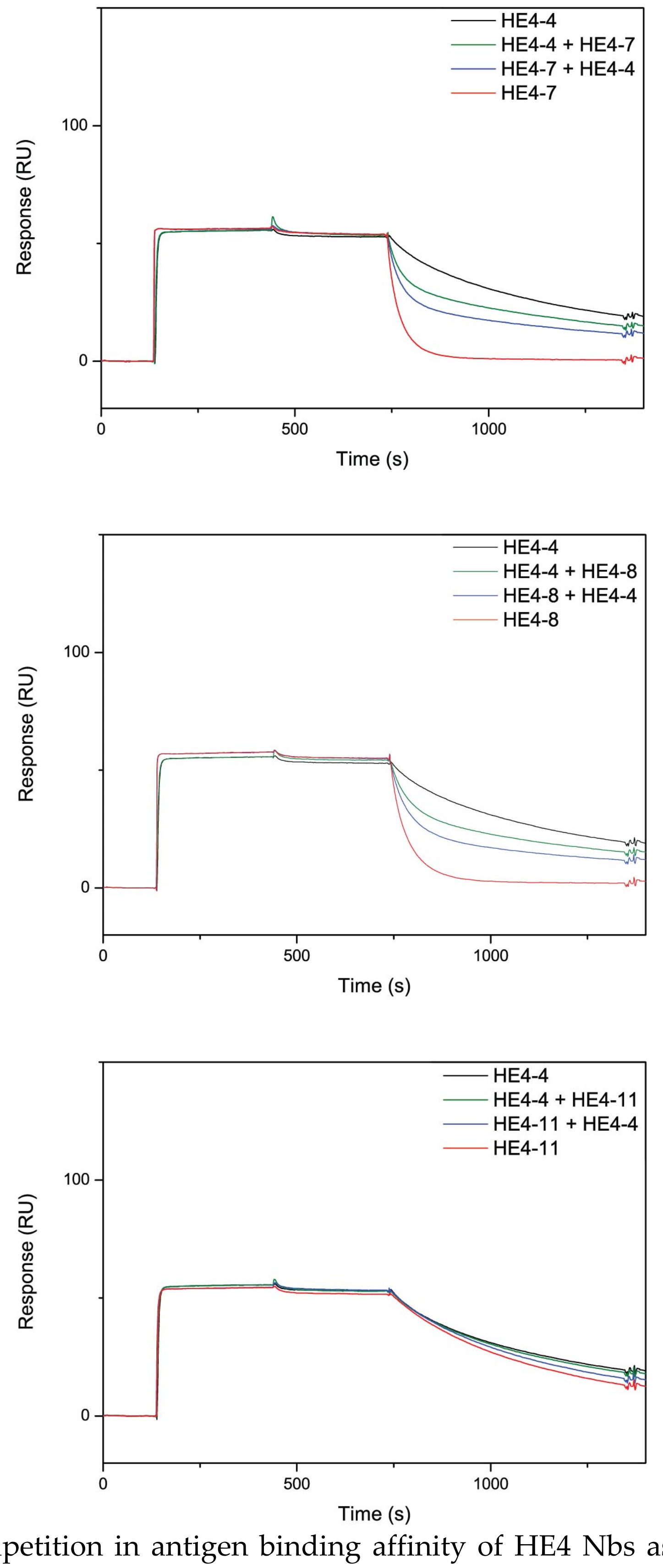

Figure S10. Competition in antigen binding affinity of HE4 Nbs as determined by epitope mapping. HE4-4 with HE4-7 (top), HE4-8 (middle) and HE4-11 (bottom).

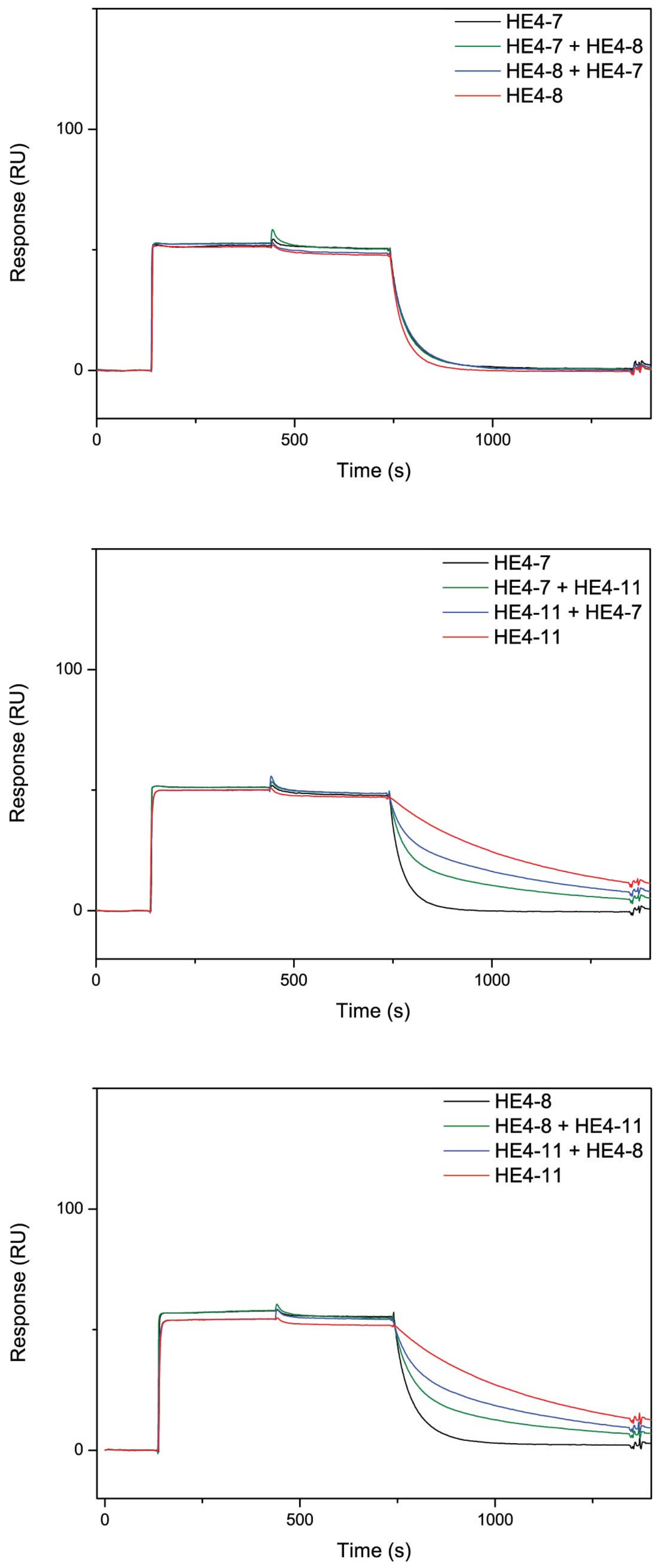

Figure S11. Competition in antigen binding affinity of HE4 Nbs as determined by epitope mapping. HE4-7 with HE4-8 (top) and HE4-11 (middle), HE4-8 with HE4-11 (bottom).

SLPI

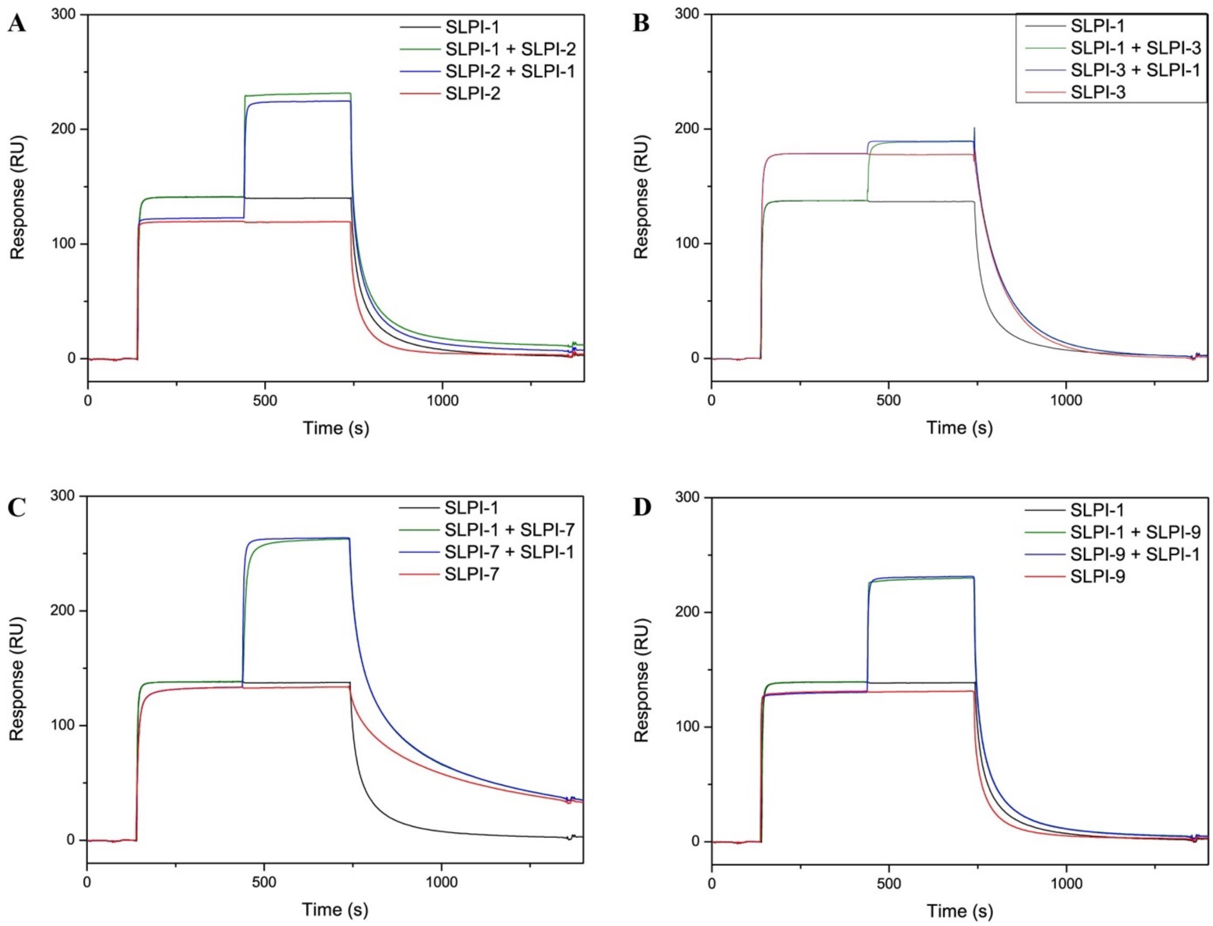

Figure S12. Competition in antigen binding affinity of SLPI Nbs as determined by epitope mapping. SLPI-1 with SLPI-2 (A), SLPI-3 (B), SLPI-7 (C) and SLPI-9 (D).

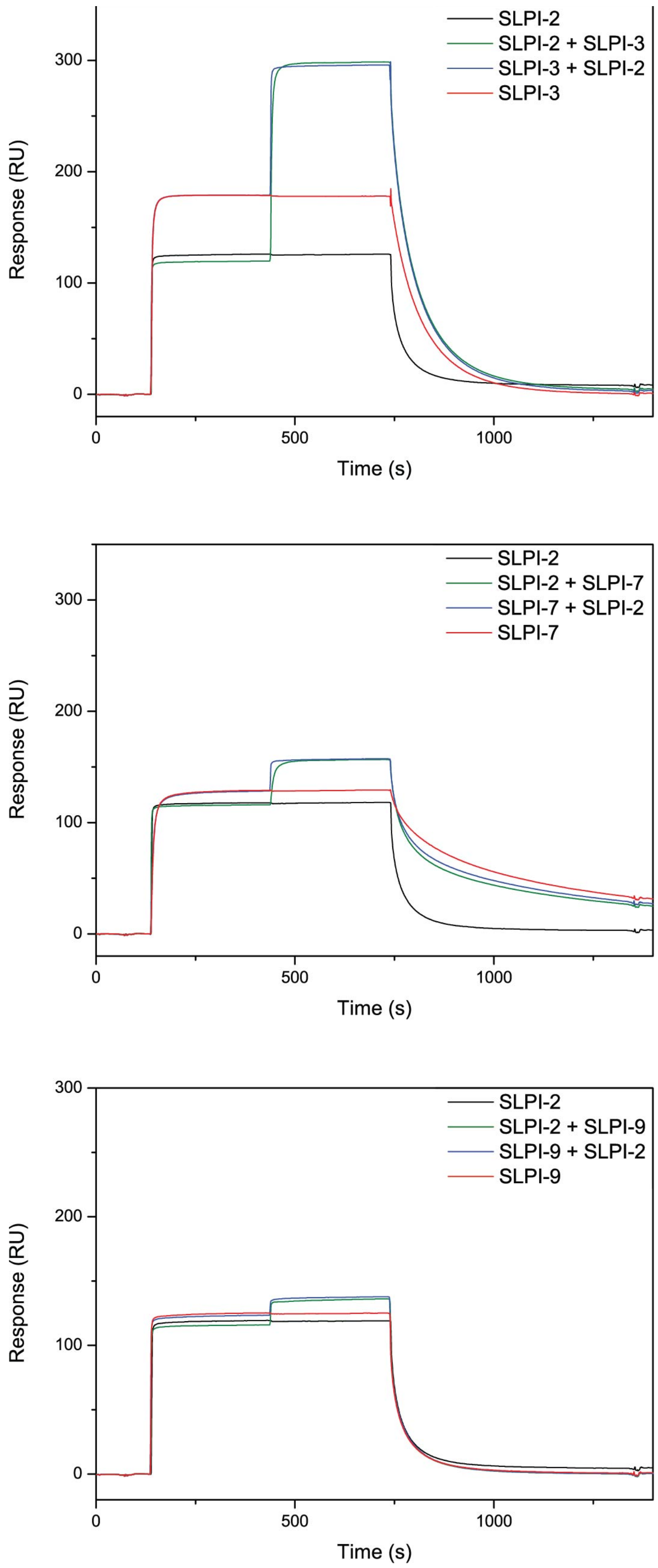

Figure S13. Competition in antigen binding affinity of SLPI Nbs as determined by epitope mapping. SLPI-2 with SLPI-3 (top), SLPI-7 (middle) and SLPI-9 (bottom).

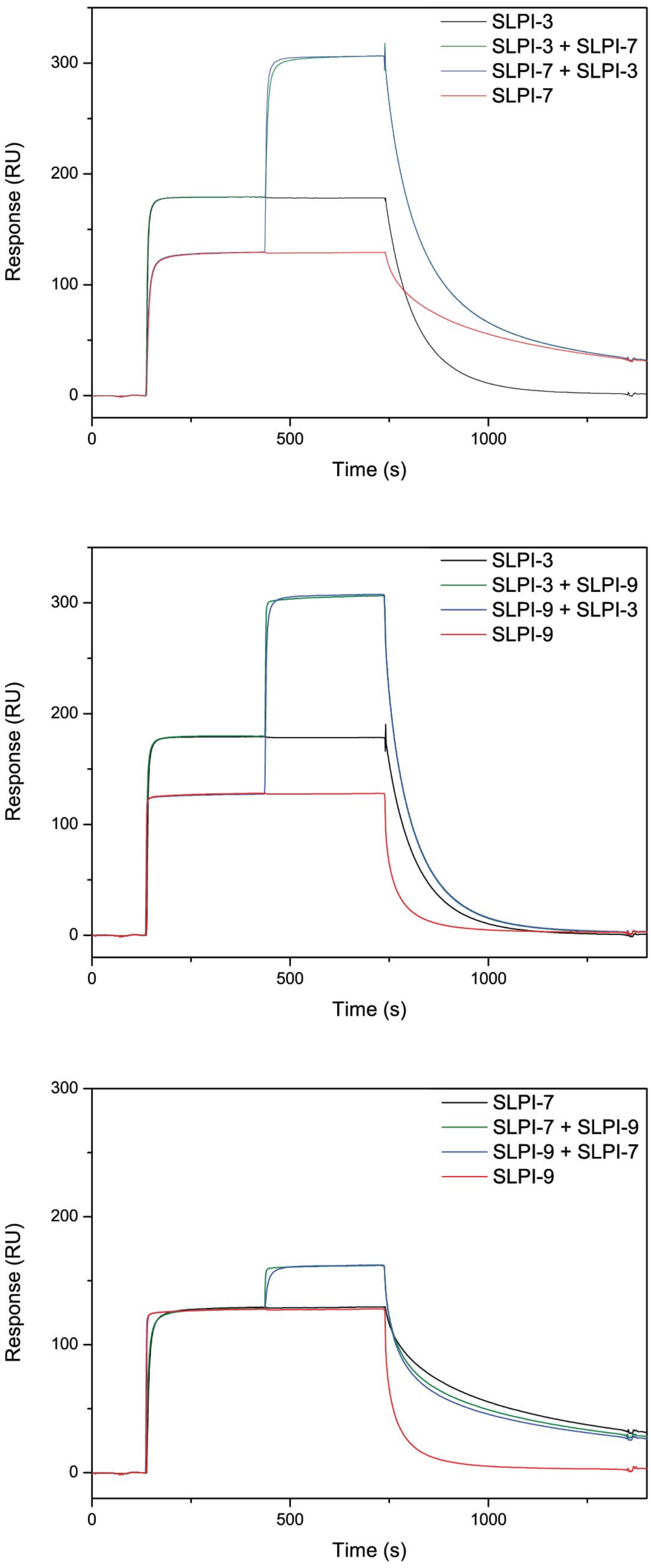

Figure S14. Competition in antigen binding affinity of SLPI Nbs as determined by epitope mapping. SLPI-3 with SLPI-7 (top) and SLPI-9 (middle), SLPI-7 with SLPI-9 (bottom).

PGRN

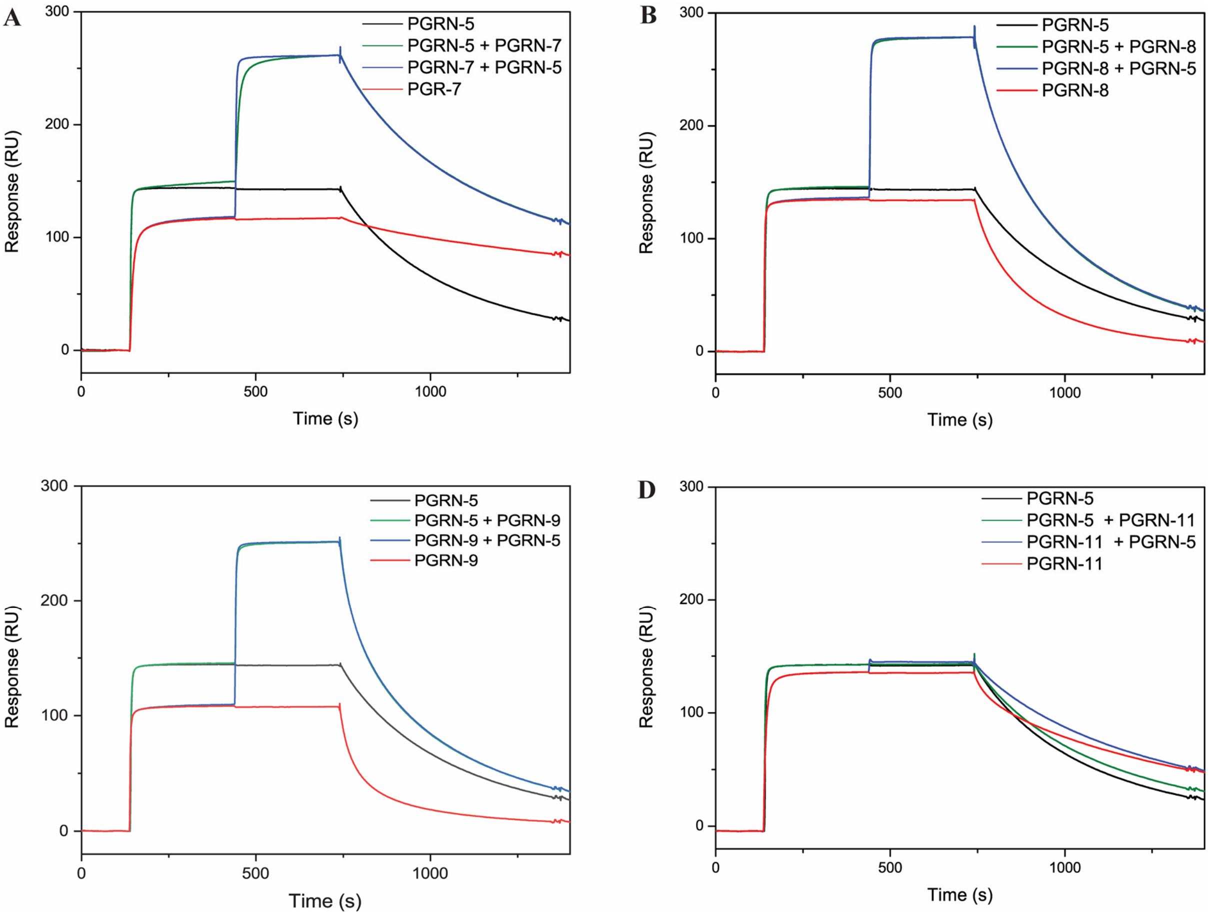

Figure S15. Competition in antigen binding affinity of PGRN Nbs as determined by epitope mapping. PGRN-5 with PGRN-7 (A), PGRN-8 (B), PGRN-9 (C) and PGRN-11 (D).

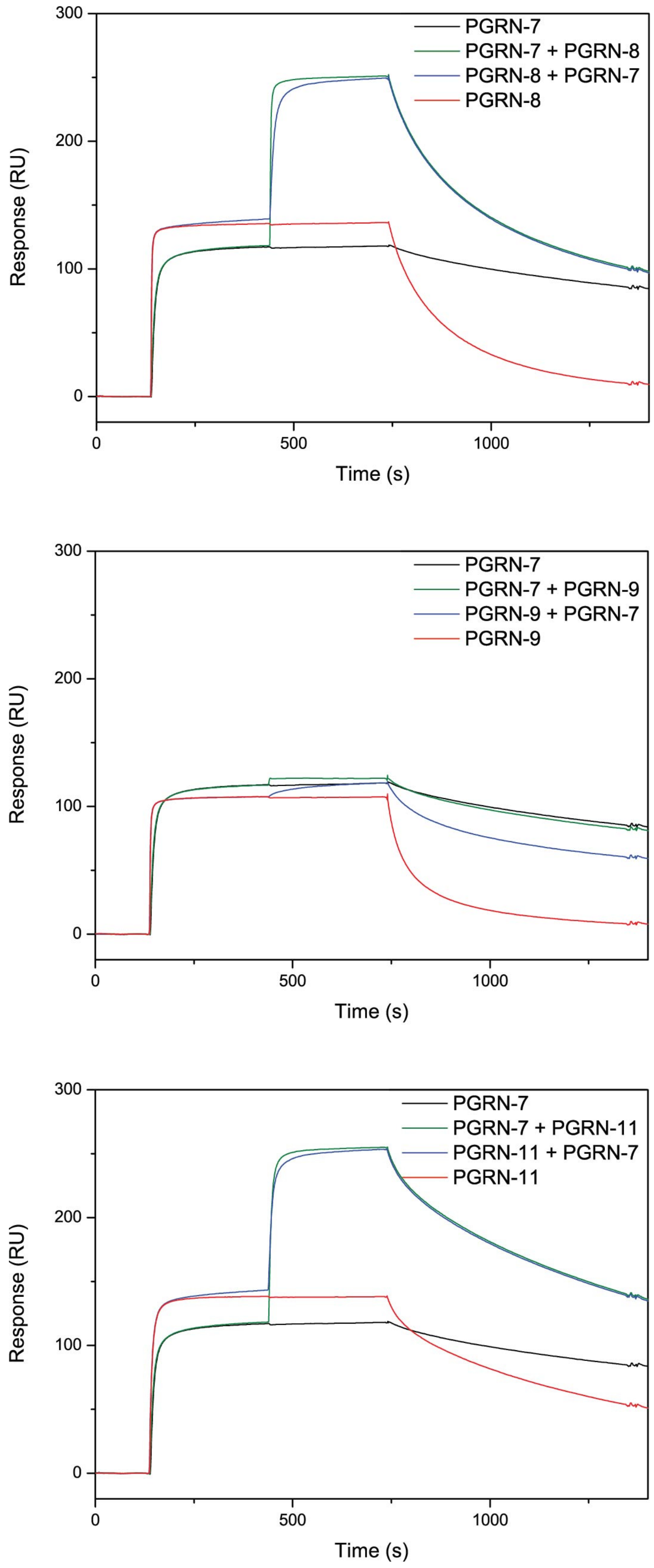

Figure S16. Competition in antigen binding affinity of PGRN Nbs as determined by epitope mapping. PGRN-7 with PGRN-8 (top), PGRN-9 (middle), and PGRN-11 (bottom).

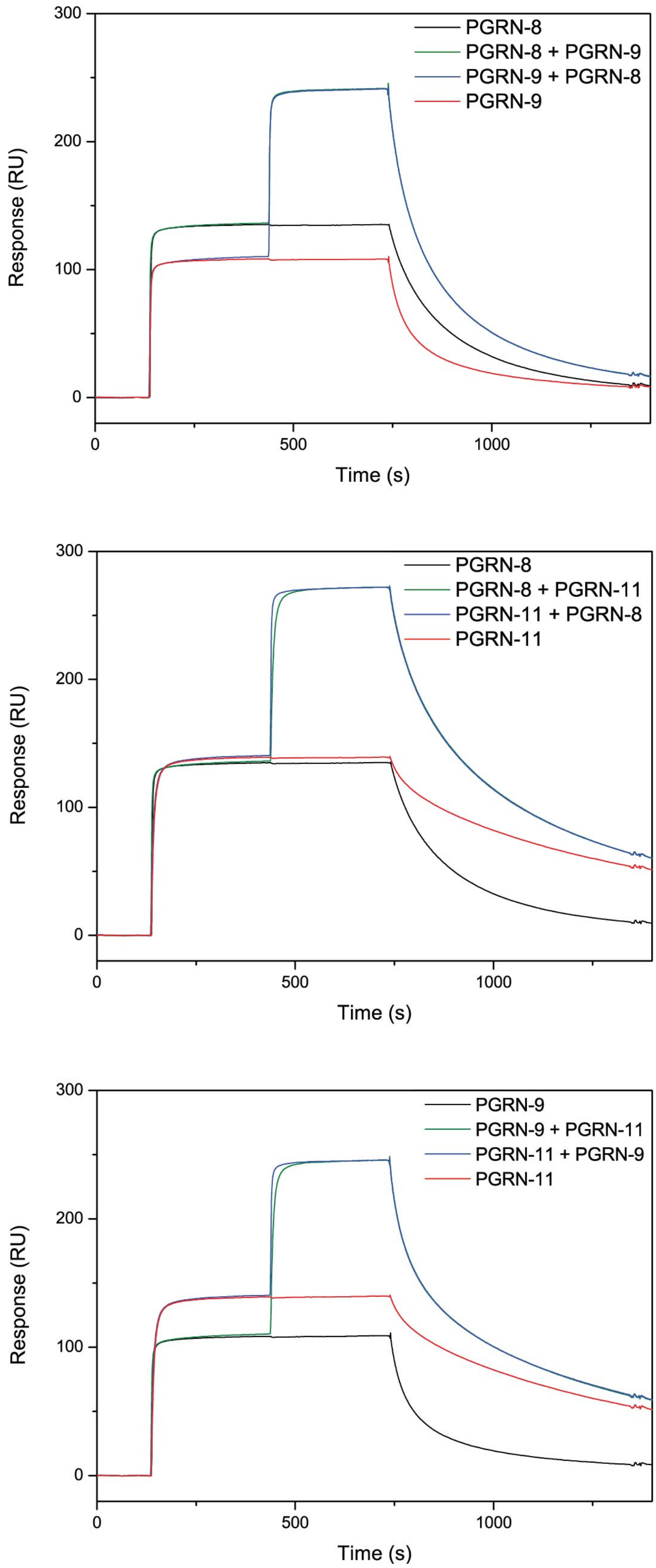

Figure S17. Competition in antigen binding affinity of PGRN Nbs as determined by epitope mapping. PGRN-8 with PGRN-9 (top), PGRN-11 (middle), PGRN-9 with PGRN-11 (bottom).

IV. Methods

1. Re-cloning of nanobody genes into pHEN6c plasmid

Nanobodies are normally expressed in the periplasm of *E. coli* to facilitate proper protein folding in the oxidizing periplasmic space. This method requires a signal leading sequence at the N-terminus of the nanobody (Nb) to facilitate the protein transport to the periplasm via the bacterial secretory pathway. In order to determine the limits of detection (LODs) using a sandwich ELISA with Streptavidin-HRP, the Nbs were site-specifically biotinylated at the C-terminus using the Sortase A protein ligation (SPL) method since modification at the N-terminus might influence the antigen binding site of the Nbs [1,2].

For re-cloning, nanobodies were re-cloned into the pHEN6c plasmid which includes the pelB sequence and His_6 tag at the N- and C-terminus respectively. The Nbs were engineered with a Leucin - Proline - Glutamic acid – Threonine – Glycine – Glycine (LPETGG) spacer between the nanobody and the His_6 tag for the proper cleavage by Sortase A. The Nb gene with the LPETGG residues was ampified by polymerase chain reaction (PCR) with Phire hot start II DNA polymerase from Thermo Fisher Scientific (Merelbeke, Belgium), a forward primer, 5'-GGT GGT CCA TGG CCC AGG TGC AG<u>C TGC AG</u>G AG-3' (PstI restriction site underlined) and a reverse primer, 5'-GGT GGT G<u>AA TT</u>C TTA GTG ATG GTG ATG GTG GTG ACC ACC AGT TTC CGG GAG TGA GGA GA-3' (EcoRI restriction site underlined). The PCR reactions were performed at 98ºC for 30 seconds and followed by 30 cycles of (98ºC for 5 seconds and 72ºC for 10 seconds). A final extension step of 10 minutes at 72ºC was run, followed by holding at 4ºC. The PCR efficiency was then checked by running a 1% agarose gel (data not shown). The pHEN6c plasmid and PCR product aimed for were then digested by restriction enzymes (PstI and EcoRI) at 37ºC for 45 minutes. The samples were subsequently run on 1% agarose gel followed by gel

extraction and purification to collect purified digested Nb gene and plasmid. The Nb gene and plasmid were then ligated to each other via a ligation reaction in the presence of T4 DNA ligase and transformed into TOP10F′ cells for cloning. For expression, the plasmid including Nb gene was re-transformed into *E. coli* WK6 cells. All Nb genes were sequenced and had accurate sequences in comparison with the DNA database. Glycerol stocks were made to preserve the strains at - 80ºC.

2. Sortase A protein ligation (SPL) mediated biotinylation

The Sortase A protein ligation mediated method was applied for the biotinylation of the Nbs. In this technique, Sortase A recognizes the LPETGG motif of the Nb and cleaves the peptide bond between the threonine and glycine resulting in a thioester intermediate containing a thioacyl function (Nb-LPET-Sortase A). An oligoglycine nucleophile then attacks the thioester intermediate to yield the transpeptidation product with the probe (Nb-biotin, in this study). The Nb-biotin was obtained by conjugating the Nb with the triglycine-lysine-biotin (GGGK-biotin) peptide from Thermo Fisher Scientific (Merelbeke, Belgium). To prepare Nb-biotin, Nb-LPETGG and Sortase A were first produced and purified. Hereto Nb-LPETGG and pET30b-7M-Sortase A (Addgene #51141), including a C-terminal His_6-tag, were expressed in the cytoplasm of BL21(DE3) *E. coli*. The growing was performed at 37°C in baffled shake flasks containing LB medium with kanamycin at 50 g/mL. Expression of the Nb was induced by adding IPTG (Duchefa biochemie, Haarlem, The Netherlands) to a final concentration of 0.5 mM and followed by culture shaken at 28°C for 6 hours. The Nbs were then extracted from the cytoplasm by incubating with B-PER extraction reagent (Thermo Fisher Scientific, Merelbeke, Belgium) and DNAse I. Hereto, the cellular pellets obtained from the 300 mL cell culture were resuspended in 6 mL B-PER buffer with 1:1000 DNAse I and pipetted up and down rigorously until the suspensions were free of clumps. The suspensions were incubated for 15

minutes at room temperature. The cytoplasmic protein extracts were harvested by centrifuging the cell suspension at 11000 g for 30 minutes at 4°C.

For purification of the Nb and sortase A, the His_6-tagged Nb-LPETGG and Sortase A were purified from the periplasmic and cytoplasmic extract by immobilized metal affinity chromatograhy (IMAC), followed by size exclusion chromatograpy (SEC), and analyzed for purity and molecular weight by SDS-PAGE. For the IMAC purification, HisPur Ni-NTA resin (Thermo Fisher Scientific) was washed twice with 50 mL PBS. After centrifugation at 300 g for 7 min, 20-25 mL of the supernatant was decanted and the HisPur Ni-NTA slurry was swirled rigorously to suspend the beads and added to the periplasmic extract. One mL of HisPur Ni-NTA suspension was sufficient to capture the His_6-tagged Nb present in a periplasmic extract derived from one liter of *E. coli* culture. The mixture was then incubated on an orbital shaking platform at 100 rpm for 1 hour at room temperature, followed by centrifuging the mixture at 300 g for 7 minutes to settle the resin. Part of the supernatant was decanted until the volume was reduced by 50%. The remaining part was then swirled and loaded into a PD-10 column (Cytiva, Hoegaarden, Belgium) by packing under gravitational force. The bed was washed with 20 mL of PBS per mL of HIS-Select suspension. The captured His_6-tagged Nbs were subsequently eluted with 5 times 1 mL of PBS containing 0.5 M imidazole and collected in 5 elution fractions for concentration measurements at 280 nm using a Nanodrop 1000 spectrophotometer.

Further purification was then performed by Size Exclusion Chromatography (SEC). A Hiload 16/600 Superdex 75 column mounted on an AKTAprime Plus was used for this process. The column was equilibrated with PBS, followed by injection of the IMAC-purified Nbs onto the column. The SEC was performed at a flow rate of 0.5 mL/min using PBS (pH 7.4) as running buffer. Fractions of the SEC showing significant UV light absorption at 280 nM were collected. Finally, the protein concentration within the SEC fractions was measured at 280 nm using the Nanodrop

1000 spectrophotometer while the purity and molecular weight of the Nbs were checked by SDS-PAGE and Coomasie staining (Figure S18). The expression yields of HE4-6-LPETGG, SLPI-1-LPETGG, PGRN-5-LPETGG are around 27 mg/L, 33 mg/L and 20 mg/L respectively.

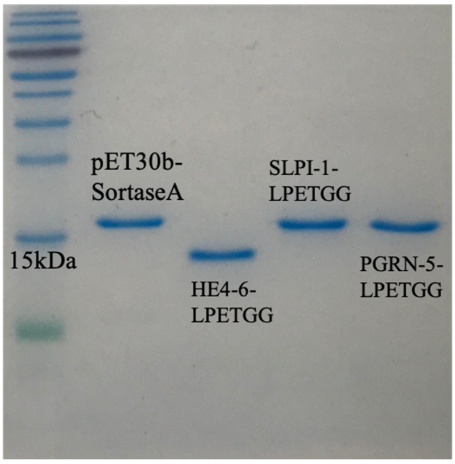

Figure S18. SDS-PAGE showing the purity and molecular weight for HE4-6-LPETGG, SLPI-1-LPETGG, PGRN-5-LPETGG and pET30b-Sortase A.

3. SDS-PAGE showing the purity and molecular weight for HE4, SLPI and PGRN

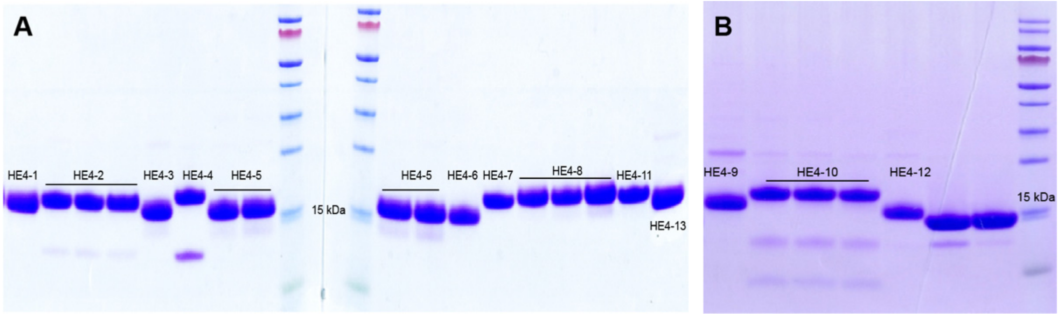

Figure S19. SDS-PAGE showing the purity and molecular weight for HE4.

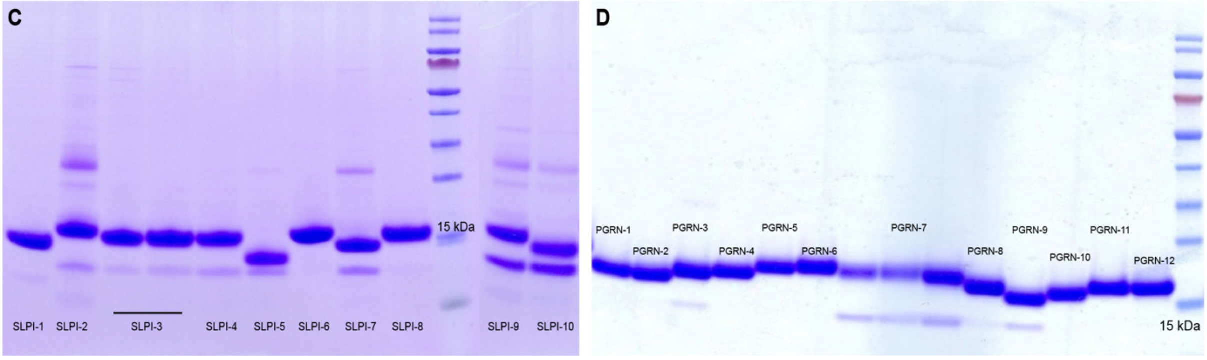

Figure S20. SDS-PAGE showing the purity and molecular weight for SLPI and PGRN.

For the Sortase A-mediated biotinylation, the reaction mixture was composed as described in Table S4 with a total volume of 300 L. The biotinylation process took place at 37°C for 24 hours. The product was then purified using a vivaspin 2 column (MWCO: 5 kDa) to remove GGGK-biotin leftover (585.49 Da) and run on SDS-PAGE gel (Fig. S21). The products might include Nb-LPETGG-His$_6$ and SortaseA-His$_6$ but only Nb-biotin can create color in ELISA reaction and the concentration was measured using a Nanodrop 1000 spectrophotometer. The Nb-biotin was then ready for use in the sandwich ELISA experiment for the LOD determination.

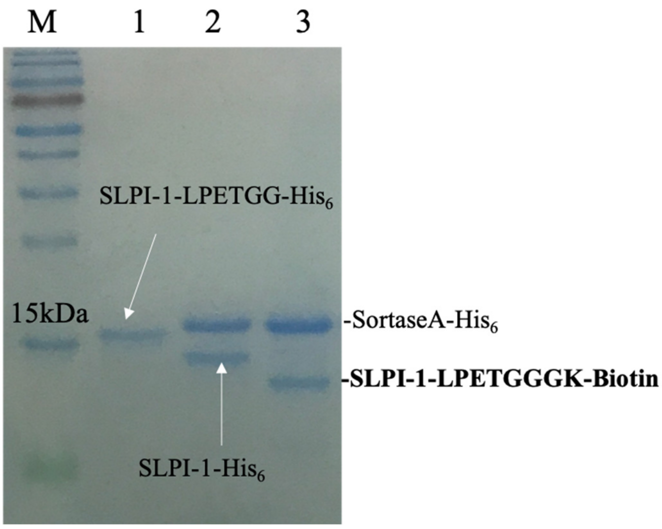

Figure S21. SDS-PAGE showing the conjugation of SLPI-1-LPETGG-His_6 to GGGK-biotin. Lane 1 – only SLPI-1-LPETGG-His_6, lane 2 – Sortase A-His_6 and SLPI-1-His_6 (negative control of SPL mediated biotinylation) and lane 3- Sortase A-His_6 and SLPI-1-LPETGGK-biotin.

Table S4. Composition of the Sortase A protein ligation mediated biotinylation reaction mixture.

No.	Stock components	Final conc.
1	GGGK-biotin	200 mM
2	β-mercaptoethanol	5 mM
3	Nb-LPETGG	50 mM
4	Sortase A	15 mM
5	10x TBS buffer, pH 7.5	1 x
6	H_2O	

Table S5. Sandwich ELISA and limit of detection (LOD) of the best performing SLPI-1 nanobody towards its respective recombinant human SLPI antigen in PBS and human serum.

	**[antigen] (pM)**	**7692.31**	**3846.15**	**1923.08**	**961.54**	**480.77**	**240.38**	**120.19**	**0**	**Slope (S)**	**y-intercept**	**R^2**
**SLPI-1** (in PBS)	**OD_1**	1.857	1.066	0.473	0.243	0.145	0.126	0.121	0.112	2.359E-04	0.068	0.994
	**OD_2**	1.482	0.917	0.386	0.193	0.126	0.123	0.131	0.110	1.878E-04	0.075	0.985
	**OD_3**	1.874	1.307	0.671	0.276	0.150	0.115	0.104	0.099	2.480E-04	0.101	0.968
	**OD_4**	1.696	1.254	0.632	0.269	0.147	0.108	0.118	0.095	2.253E-04	0.110	0.955
	**$OD_{average}$**	1.727	1.136	0.541	0.245	0.142	0.118	0.119	**0.104**	**2.242E-04**	**0.089**	0.981
	**Stand. Dev.**	0.182	0.179	0.134	0.038	0.011	0.008	0.011	**0.008**			
	**yLOD**	yLOD = mean of blank + 3 * stdv of blank										
	**LOD (pM)**	LOD (pM) = (yLOD – y-intercept of $OD_{average}$)/ Slope of $OD_{average}$										
**SLPI-1** (in human serum)	**[antigen] (pM)**	**7692.31**	**3846.15**	**1923.08**	**961.54**	**480.77**	**240.38**	**120.19**	**0**	**Slope (S)**	**y-intercept**	**R^2**
	**OD_1**	1.625	1.073	0.719	0.493	0.366	0.338	0.288	0.281	1.786E-04	0.307	0.989
	**OD_2**	1.422	1.124	0.804	0.515	0.421	0.336	0.337	0.292	1.529E-04	0.365	0.938
	**OD_3**	1.407	1.078	0.718	0.494	0.418	0.308	0.306	0.267	1.528E-04	0.333	0.956
	**OD_4**	1.399	1.375	0.898	0.624	0.462	0.346	0.334	0.337	1.548E-04	0.427	0.832
	**$OD_{average}$**	1.463	1.163	0.785	0.532	0.417	0.332	0.316	0.294	**1.598E-04**	0.358	0.942

	**Stand. Dev.**	0.108	0.144	0.086	0.062	0.039	0.017	0.017	0.030			
	**yLOD**	yLOD = mean of blank + 3 * stdv of blank										
	**LOD (pM)**	LOD (pM) = (yLOD – y-intercept of $OD_{average}$)/ Slope of $OD_{average}$										

Table S6. Sandwich ELISA and limit of detection (LOD) of the best performing PGRN-5 nanobody towards its respective recombinant human PGRN antigen in PBS and human serum.

	**[antigen] (pM)**	**1111.11**	**555.56**	**277.78**	**138.89**	**69.44**	**34.72**	**17.36**	**0**	**Slope (S)**	**y-intercept**	**R^2**
	**OD_1**	1.465	0.875	0.706	0.468	0.384	0.306	0.314	0.315	1.046E-0	0.316	0.989
	**OD_2**	1.319	0.882	0.712	0.468	0.362	0.325	0.330	0.320	9.247E-04	0.335	0.977
**PGRN-5** (in PBS)	**OD_3**	1.339	0.688	0.528	0.422	0.359	0.392	0.325	0.320	8.805E-04	0.304	0.978
	**OD_4**	1.389	0.644	0.621	0.456	0.343	0.401	0.371	0.361	8.833E-04	0.330	0.947
	**$OD_{average}$**	1.378	0.772	0.642	0.454	0.362	0.356	0.335	**0.329**	**9.336E-04**	**0.321**	0.990
	**Stand. Dev.**	0.065	0.124	0.086	0.022	0.017	0.048	0.025	**0.021**			
	**yLOD**	yLOD = mean of blank + 3 * stdv of blank										
	**LOD (pM)**	LOD (pM) = (yLOD – y-intercept of $OD_{average}$)/ Slope of $OD_{average}$										

PGRN-5 (in human serum)	[antigen] (pM)	1111.11	555.56	277.78	138.89	69.44	34.72	17.36	0	Slope (S)	y-intercept	R^2
	**OD_1**	1.268	0.715	0.601	0.536	0.430	0.445	0.487	0.498	7.019E-04	0.429	0.955
	**OD_2**	1.207	0.761	0.548	0.421	0.447	0.455	0.392	0.422	7.183E-04	0.384	0.982
	**OD_3**	1.072	0.718	0.603	0.548	0.460	0.502	0.435	0.435	5.538E-04	0.444	0.986
	**OD_4**	0.958	0.685	0.587	0.536	0.519	0.475	0.509	0.444	4.285E-04	0.471	0.985
	**$OD_{average}$**	1.126	0.720	0.585	0.510	0.464	0.469	0.456	0.450	**6.006E-04**	0.432	0.990
	**Stand. Dev.**	0.139	0.031	0.026	0.060	0.039	0.025	0.053	0.033			
	**yLOD**	yLOD = mean of blank + 3 * stdv of blank										
	**LOD (pM)**	LOD (pM) = (yLOD – y-intercept of $OD_{average}$)/ Slope of $OD_{average}$										

Table S7. Sandwich ELISA and limit of detection (LOD) of the best performing HE4-6 nanobody towards its respective recombinant human HE4 antigen in PBS and human serum

**HE4-6** (in PBS)	[antigen] (pM)	4000	2000	1000	500	250	125	62.5	0	Slope (S)	y-intercept	R^2
	**OD_1**	1.408	0.861	0.761	0.499	0.324	0.231	0.211	0.227	2.973E-04	0.270	0.951

	**OD_2**	1.258	0.829	0.683	0.624	0.266	0.203	0.197	0.198	2.649E-04	0.269	0.900
	**OD_3**	1.281	1.032	0.614	0.386	0.263	0.177	0.208	0.155	2.981E-04	0.219	0.933
	**OD_4**	1.315	0.900	0.532	0.318	0.253	0.200	0.186	0.154	2.990E-04	0.186	0.982
	**$OD_{average}$**	1.316	0.906	0.648	0.457	0.277	0.203	0.201	**0.184**	**2.898E-04**	**0.236**	0.961
	**Stand. Dev.**	0.066	0.089	0.098	0.134	0.032	0.022	0.011	**0.036**			
	**yLOD**	yLOD = mean of blank + 3 * stdv of blank										
	**LOD (pM)**	LOD (pM) = (yLOD – y-intercept of $OD_{average}$)/ Slope of $OD_{average}$										
**HE4-6** (in human serum)	**[antigen] (pM)**	**4000**	**2000**	**1000**	**500**	**250**	**125**	**62.5**	**0**	**Slope (S)**	**y-intercept**	**R^2**
	**OD_1**	0.938	0.541	0.406	0.261	0.207	0.177	0.166	0.146	1.958E-04	0.161	0.994
	**OD_2**	0.928	0.527	0.402	0.273	0.206	0.184	0.165	0.148	1.916E-04	0.164	0.994
	**OD_3**	0.854	0.608	0.400	0.302	0.219	0.178	0.183	0.159	1.777E-04	0.187	0.976
	**OD_4**	0.940	0.537	0.342	0.257	0.200	0.165	0.162	0.140	1.979E-04	0.146	0.999
	**$OD_{average}$**	0.915	0.553	0.388	0.273	0.208	0.176	0.169	0.148	**1.908E-04**	0.165	0.996
	**Stand. Dev.**	0.041	0.037	0.030	0.020	0.008	0.008	0.009	0.008			
	**yLOD**	yLOD = mean of blank + 3 * stdv of blank										
	**LOD (pM)**	LOD (pM) = (yLOD – y-intercept of $OD_{average}$)/ Slope of $OD_{average}$										

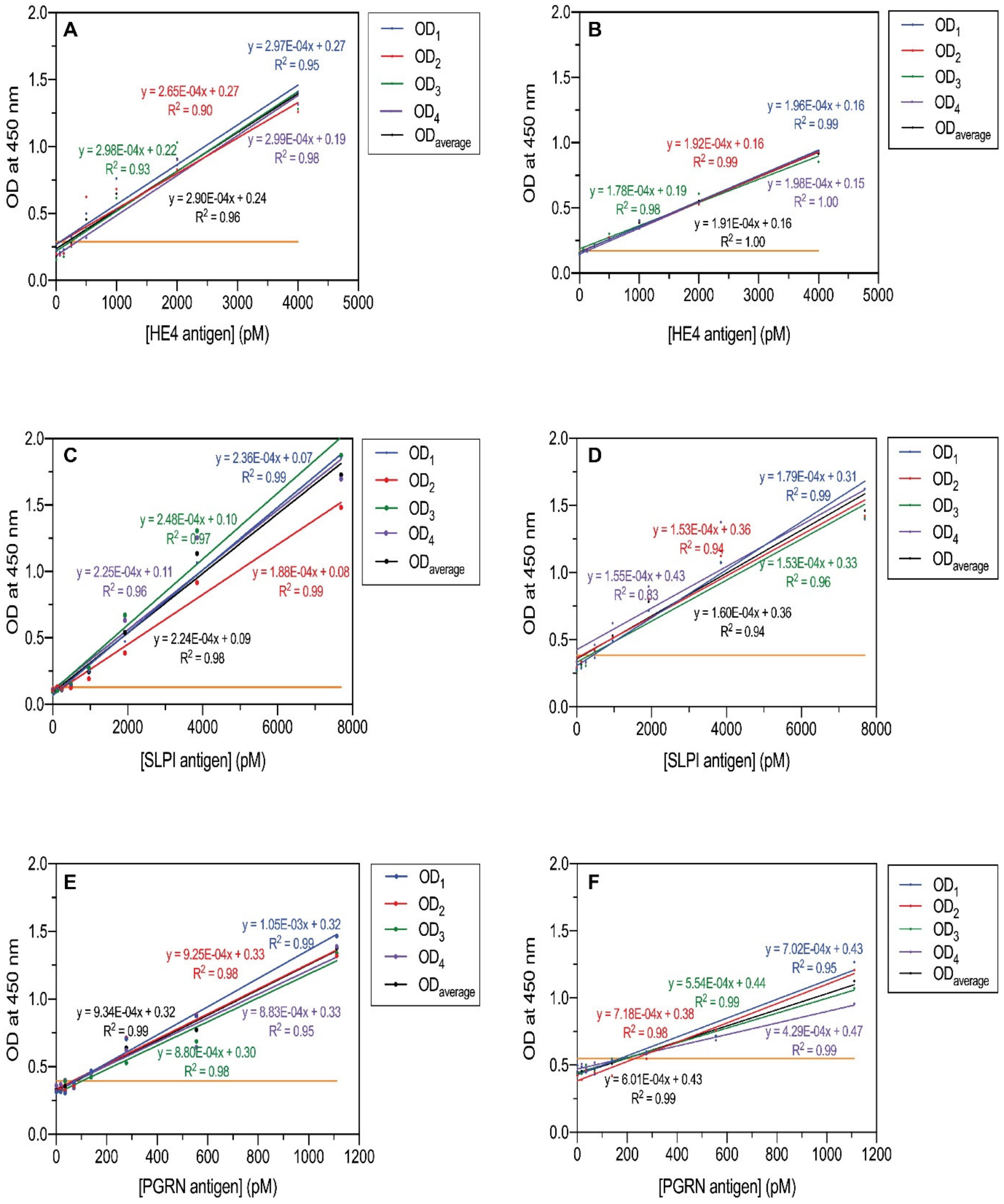

Figure S22. Sandwich ELISA plots showing the detection results of the recombinant human antigens HE4, SLPI and PGRN by the respective best performing nanobodies HE4-6 in PBS (A) and serum (B); SLPI-1 in PBS (C) and serum (D); and PGRN-5 in PBS (E) and serum (F). The yellow line indicates the LOD.

1. Graulus G-J, Ta DT, Tran H, *et al.* Site-Selective Functionalization of Nanobodies Using Intein-Mediated Protein Ligation for Innovative Bioconjugation. In: *Bioconjugation Methods and Protocols*. Massa S, Devoogdt N (Eds.), Humana Press (2019).
2. Billen B, Vincke C, Hansen R, *et al.* Cytoplasmic versus periplasmic expression of site-specifically and bioorthogonally functionalized nanobodies using expressed protein ligation. *Protein Expr. Purif.* [Internet]. 133, 25–34 (2017). Available from: http://www.sciencedirect.com/science/article/pii/S1046592816301917.
